# Feasibility and Outcome of PSMA-PET-Based Dose-Escalated Salvage Radiotherapy *Versus* Conventional Salvage Radiotherapy for Patients With Recurrent Prostate Cancer

**DOI:** 10.3389/fonc.2021.715020

**Published:** 2021-07-30

**Authors:** Marco M. E. Vogel, Sabrina Dewes, Eva K. Sage, Michal Devecka, Kerstin A. Eitz, Jürgen E. Gschwend, Matthias Eiber, Stephanie E. Combs, Kilian Schiller

**Affiliations:** ^1^Department of Radiation Oncology, Klinikum rechts der Isar, Technical University of Munich (TUM), Munich, Germany; ^2^Institute for Radiation Medicine (IRM), Department of Radiation Sciences (DRS), Helmholtz Zentrum München, Neuherberg, Germany; ^3^Deutsches Konsortium für Translationale Krebsforschung (DKTK), Partner Site Munich, Munich, Germany; ^4^Department of Urology, Klinikum rechts der Isar, Technical University of Munich (TUM), Munich, Germany; ^5^Department of Nuclear Medicine, Klinikum rechts der Isar, Technical University of Munich (TUM), Munich, Germany

**Keywords:** simultaneous-integrated boost, relapse, positron emission tomography, prostate-specific membrane antigen, side effects, disease-free survival

## Abstract

**Introduction:**

Prostate-specific membrane antigen-positron emission tomography-(PSMA-PET) imaging facilitates dose-escalated salvage radiotherapy (DE-SRT) with simultaneous-integrated boost (SIB) for PET-positive lesions in patients with prostate cancer (PC). Therefore, we aimed to compare toxicity rates of DE-SRT with SIB to conventional SRT (C-SRT) without SIB and to report outcome.

**Materials and Methods:**

We evaluated 199 patients who were treated with SRT between June 2014 and June 2020. 101 patients received DE-SRT with SIB for PET-positive local recurrence and/or PET-positive lymph nodes. 98 patients were treated with C-SRT to the prostate bed +/− elective pelvic lymphatic pathways without SIB. All patients received PSMA-PET imaging prior to DE-SRT ([68Ga]PSMA-11: 45.5%; [18F]-labeled PSMA: 54.5%). Toxicity rates for early (<6 months) and late (>6 months) gastrointestinal (GI) toxicities rectal bleeding, proctitis, stool incontinence, and genitourinary (GU) toxicities hematuria, cystitis, urine incontinence, urinary obstruction, and erectile dysfunction were assessed. Further, we analyzed the outcome with disease-free survival (DFS) and prostate-specific antigen (PSA) response.

**Results:**

The overall toxicity rates for early GI (C-SRT: 2.1%, DE-SRT: 1.0%) and late GI (C-SRT: 1.4%, DE-SRT: 5.3%) toxicities ≥ grade 2 were similar. Early GU (C-SRT: 2.1%, DE-SRT: 3.0%) and late GU (C-SRT: 11.0%, DE-SRT: 14.7%) toxicities ≥ grade 2 were comparable, as well. Early and late toxicity rates did not differ significantly between DE-SRT *versus* C-SRT in all subcategories (p>0.05). PSA response (PSA ≤0.2 ng/ml) in the overall group of patients with DE-SRT was 75.0% and 86.4% at first and last follow-up, respectively.

**Conclusion:**

DE-SRT showed no significantly increased toxicity rates compared with C-SRT and thus is feasible. The outcome of DE-SRT showed good results. Therefore, DE-SRT with a PSMA-PET-based SIB can be considered for the personalized treatment in patients with recurrent PC.

## Introduction

Salvage radiotherapy (SRT) is an integral part of prostate cancer (PC) treatment. Approximately one third to one half of the patients undergoing radical prostatectomy (RP) will develop a biochemical relapse (1). Recently, three randomized controlled trials evaluated observation with SRT *versus* adjuvant RT (2–4). The data suggest that observation with SRT can be considered as the standard treatment option for most patients after RP. However, especially for patients with high-risk features adjuvant RT should be discussed as well.

With the introduction of the prostate-specific membrane antigen-positron emission tomography (PSMA-PET) imaging, it quickly became a valid diagnostic tool for patients with PC relapse. PSMA tracers allow for detection rates of 58% at prostate-specific antigen (PSA) levels as low as 0.2 to 1.0 ng/ml for [68Ga]-labeled PSMA, increasing with higher PSA values (5).

Whereas in the past, the radiation oncologist had to treat the prostate bed (PB) and/or the elective pelvic lymph nodes (ePLNs) in cases of SRT mostly without an imaging correlate and based on statistical probabilities, today, RT of the tumor volume visualized by PSMA-PET is possible. The precise imaging allows for treatment of the macroscopic disease [local recurrence or pelvic lymph nodes (LNs)] with higher doses than the elective PB or ePLNs. With modern intensity-modulated RT (IMRT) a simultaneous-integrated boost (SIB) is possible, without prolonging the total treatment time.

However, it remains unknown, if side effects of PSMA-PET-based dose-escalated SRT (DE-SRT) with SIB are increased compared with conventional SRT (C-SRT) without SIB. Therefore, this study aims to compare toxicity of DE-SRT *versus* C-SRT. Further, we report the outcome of patients receiving PSMA-PET-based DE-SRT.

## Materials And Methods

### Patients

We screened 256 patients who were treated between June 2014 and June 2020 at the University Hospital of the Technical University of Munich (TUM). We included patients with relapse after RP who received either DE-SRT with SIB for PET-positive local recurrence or LNs as well as C-SRT without SIB. Patients had a post-RP PSA nadir of <0.1 ng/ml. We excluded patients due to distant metastases or 3-dimensional RT, as well as the use of Choline-PET instead of PSMA-PET or sequential boost techniques. Further, we excluded patients if they showed PET-positive lesions, but no dose escalation was performed. In line with the recent guidelines (6, 7) and to ensure comparability, we excluded patients with doses of EQD2 (1.5 Gy) < 66 Gy to the PB. Patients without follow-up were excluded as well. Analysis was conducted retrospectively and was part of the SIMBA (Simultaneous-Integrated Boost in Salvage Radiotherapy for Patients With Recurrent Prostate Cancer) study. The institutional review board of the Technical University of Munich (TUM) approved the study (No. 564/19-S).

### PSMA-PET Imaging

Before DE-SRT, each patient received PET imaging with [68Ga]PSMA-11 (8) or a [18F]-labeled PSMA-ligand ([18F]PSMA-1007 (9), [18F]rhPSMA-7 (10), or [18F]rhPSMA-7.3 (11)). PET acquisition was performed according to the joint EANM and SNMMI guidelines (12). Imaging was acquired in conjunction with either a diagnostic computed tomography (CT) or magnetic resonance imaging (MRI). Intravenous and oral contrast agents were used if the patient had no contraindications both for PET/CT and PET/MRI. When possible, furosemide 20 mg was given to reduce tracer collection in the urinary tract system. One specialist in nuclear medicine and one radiologist or a dual boarded nuclear medicine physician/radiologist interpreted the scans. Focal tracer uptake higher than the surrounding background and not associated with physiologic uptake was considered as suspect.

### Radiotherapy

RT was performed with intensity-modulated RT (IMRT) as volumetric arc therapy (VMAT) or helical IMRT. Planning CT and RT were performed with a reproducible comfortably filled bladder and empty rectum. We performed image-guided RT (IGRT) with daily online imaging. Target delineation was conducted using the RTOG (13) or EORTC (14) guideline. Planning target volume (PTV) of the SIBs were generated with an additional margin of 5 to 10 mm to the gross tumor volume (GTV). Indication for additive androgen deprivation therapy was discussed in a multidisciplinary tumor board and recommended thereafter to the patient. When organ at risk constraints allowed, we used the following dose concept: Overall, the PB was irradiated with a total of 68 Gy in 2 Gy single doses (34 fractions). The ePLNs were treated with 50.4 Gy in 1.8 Gy single doses (28 fractions). When patients received RT to the PB and ePLNs we treated the PB for 28 fractions up to 56 Gy and the ePLNs up to 50.4 Gy continuing with the PB only up to the total dose of 68 Gy. In the DE-SRT group, we treated the patients with an additional SIB to the PET-positive areas (local recurrence and/or LNs). Then the PB was irradiated with 68 Gy in 2 Gy single doses (34 fractions) and a SIB to the local recurrence with 76.5 Gy in 2.25 Gy doses (34 fractions). ePLNs were treated with 50.4 Gy in 1.8 Gy doses (28 fractions) and a SIB to PET positive areas with 58.8 Gy in 2.1 Gy doses (28 fractions) or 61.6 Gy in 2.2 Gy doses (28 fractions). When patients received RT to the PB and ePLNs with SIB we treated the PB and the ePLNs for 28 fractions continuing with the PB only for a total of 34 fractions. However, changes to the total doses of PB, ePLNs, and SIBs were possible and at the discretion of the treating radiation oncologist.

### Toxicity

Toxicity of SRT was assessed using the Common Terminology Criteria for Adverse Events (CTCAE) version 5 (15). Follow-up was conducted according to our institutional protocol. First follow-up was performed 4 to 6 weeks after termination of RT, thereafter time intervals increased to 3 and 6 months, before continuing with yearly visits. Outpatient urologic aftercare including PSA tests were recommended every 3 months for the first 2 years, every 6 months for the following 2 years continuing with annual appointments. Side effects before 6 months were classified as early/acute toxicity, whereas late/chronic toxicity was defined as side effects after 6 months. Only newly occurred or worsened side effects were defined as related to RT.

### Outcome

We defined PSA response after SRT as a PSA value below or equal 0.2 ng/ml. Disease-free survival (DFS) was defined as either PSA progression (PSA nadir + 0.2 ng/ml and one confirmation value), local relapse, occurrence of metastasis or change/initiation of ADT.

### Statistics

To compare baseline characteristics and toxicity in both groups we used a Pearson’s chi-square test or an independent-samples median test. Patients without follow-up data were excluded from the evaluation of the respective toxicity endpoint. Toxicity rates were compared by Pearson’s chi-square test. For the analysis of DFS, we used Cox regression analysis adjusted for the use of additive ADT.

The median PSA before RT was significantly different. To ensure comparability, we only included patients in the outcome analysis whose PSA levels met the common definition of a relapse of >0.2 ng/ml (16) (n=148). Median time between ADT and last follow-up was 7 months (range: 0–51 months). Since ADT influences the PSA response, we excluded patients with admission of ADT in follow-up after the termination of additive ADT from evaluation of the PSA response. To compare doses with different fractionation schemes, we used the equivalent dose in 2 Gy fractions with an alpha/beta ratio of 1.5 Gy (EQD2, 1.5 Gy). Wherever possible, we report the EQD2 (1.5 Gy). All statistical analyses were performed with SPSS version 21 (IBM, Armonk, USA). A p-value <0.05 was considered as statistically significant.

## Results

After screening, we evaluated 199 patients with a median age of 71.0 years (range, 49.0–82.0 years). Median follow-up was 13.6 months (range, 0.4–70.0 months). Complete patient characteristics are shown in **Table 1**.

**Table 1:** Patient characteristics.

	All patients, n = 199 (%)	C-SRT, n = 98 (%)	DE-SRT, n = 101 (%)	*p*
**Age [Years]**	71.0 (range: 49.0-82.0)	69.0 (range: 52.0-82.0)	72.0 (range: 49.0-82.0)	0.07
**Treatment Fields**				
PB	85 (42.7%)	85 (86.7%)	N./a.	*N./a.*
PB + ePLNs	13 (6.5%)	13 (13.3%)	N./a.	
PB/SIB	55 (27.7%)	*N./a.*	55 (54.5%)	
PB/SIB + ePLNs	11 (5.5%)	*N./a.*	11 (10.9%)	
PB + ePLNs/SIB	16 (8.1%)	*N./a.*	16 (15.8%)	
PB/SIB + ePLNs/SIB	15 (7.5%)	*N./a.*	15 (14.8%)	
ePLNs/SIB	4 (2.0%)	*N./a.*	4 (4.0%)	
**Postoperative Tumor Classification**				
pT1c	1 (0.5%)	1 (1.0%)	0 (0.0%)	0.89
pT2	5 (2.5%)	2 (2.0%)	3 (3.0%)	
pT2a	10 (5.1%)	3 (3.1%)	7 (6.9%)	
pT2b	5 (2.5%)	3 (3.1%)	2 (2.0%)	
pT2c	78 (39.2%)	40 (40.8%)	38 (37.6%)	
pT3	2 (1.0%)	1 (1.0%)	1 (1.0%)	
pT3a	52 (26.1%)	28 (28.6%)	24 (23.7%)	
pT3b	41 (20.6%)	19 (19.4%)	22 (21.8%)	
pT4	2 (1.0%)	1 (1.0%)	1 (1.0%)	
Missing	3 (1.5%)	0 (0.0%)	3 (3.0%)	
**Postoperative Nodal Status**				
Negative (pN0)	165 (82.9%)	84 (85.7%)	81 (80.2%)	0.65
Positive (pN1)	26 (13.1%)	12 (12.3%)	14 (13.9%)	
Unknown (pNx)	6 (3.0%)	2 (2.0%)	4 (3.9%)	
Missing	2 (1.0%)	0 (0.0%)	2 (2.0%)	
**Postoperative Surgical Margin**				
Negative (R0)	142 (71.4%)	71 (72.5%)	71 (70.3%)	0.10
Positive (R1)	45 (22.6%)	26 (26.5%)	19 (18.8%)	
Unknown (Rx)	7 (3.5%)	1 (1.0%)	6 (5.9%)	
Missing	5 (2.5%)	0 (0.0%)	5 (5.0%)	
**Gleason Score**				
ISUP Group 1 (≤6)	12 (6.0%)	9 (9.2%)	3 (3.0%)	0.10
ISUP Group 2 (3 + 4 = 7)	80 (40.2%)	41 (41.8%)	39 (38.6%)	
ISUP Group 3 (4 + 3 = 7)	52 (26.1%)	20 (20.4%)	32 (31.7%)	
ISUP Group 4 (8)	19 (9.6%)	12 (12.3%)	7 (6.9%)	
ISUP Group 5 (9-10)	30 (15.1%)	14 (14.3%)	16 (15.8%)	
Gleason Score 7 without specification	2 (1.0%)	2 (2.0%)	0 (0.0%)	
Missing	4 (2.0%)	0 (0.0%)	4 (4.0%)	
**Median time between resection and RT [Months]**	37.60 (range: 3.10-293.30)	26.05 (range 3.10-166.30)	51.10 (range:4.60-293.30)	<0.001*
**PSA at recurrence [ng/ml]**	0.32 (range: 0.02-22.00)	0.21 (range: 0.02-5.64)	0.45 (range:0.02-22.00)	<0.01*
≤0.5 ng/ml	145 (72.9%)	90 (91.8%)	55 (54.5%)	<0.001*
0.5-2.0 ng/ml	37 (18.6%)	5 (5.1%)	32 (31.7%)	
>2.0 ng/ml	17 (8.5%)	3 (3.1%)	14 (13.8%)	
**PSMA-PET Imaging**				
[^68^Ga]PSMA-11	70 (35.2%)	24 (24.5%)	46 (45.5%)	<0.001*
[^18^F]rhPSMA-7	28 (14.1%)	6 (6.1%)	22 (21.8%)	
[^18^F]rhPSMA-7.3	36 (18.1%)	11 (11.2%)	25 (24.8%)	
[^18^F]PSMA-1007	10 (5.0%)	2 (2.1%)	8 (7.9%)	
No PET	55 (27.6%)	55 (56.1%)	0 (0.0%)	
**Results PSMA-PET Imaging**				
Local recurrence (rcT+)	58 (57.4%)	0 (0.0%)	58 (57.4%)	*N./a.*
Lymph node metastasis (rcN+)	18 (17.8%)	0 (0.0%)	18 (17.8%)	
Local recurrence and lymph node metastasis (rcT+ and rcN+)	25 (24.8%)	0 (0.0%)	25 (24.8%)	
**Additive ADT**				
Yes	40 (20.1%)	12 (12.2%)	28 (27.7%)	0.006*
No	159 (79.9%)	86 (87.8%)	73 (72.3%)	
**Median Follow-Up [Months]**	13.6 (range: 0.4-70.0)	18.9 (range: 0.4-70.0)	10.7 (range: 0.7-59.4)	0.14

C-SRT, conventional salvage radiotherapy; DE-SRT, dose-escalated salvage radiotherapy; PB, prostate bed; SIB, simultaneous-integrated boost; ePLNs, elective pelvic lymph nodes; N./a., not applicable; ISUP, International Society of Urological Pathology; PSMA, prostate-specific membrane antigen; RT, radiotherapy; PET, positron emission tomography; Ga, Gallium; F, flour; ADT, androgen deprivation therapy; *significant result.

Patients were treated between 06/2014 und 06/2020 with the median doses shown in **Table 2**.

**Table 2:** Radiation doses for conventional salvage radiotherapy (C-SRT) and dose-escalated salvage radiotherapy (DE-SRT).

	C-SRT		DE-SRT	
	Median total dose [Gy]	Single dose [Gy]	Median total dose [Gy]	Single dose [Gy]
**PB**	68.00 (range: 66.00-70.00)	2.00 (range: 2.00-2.00)	68.00 (range, 68.00–70.00)	2.00 (range, 1.80–2.00)
**Elective pelvic LNs**	50.40 (range: 50.40-50.40)	1.80 (range: 1.80-1.80)	50.40 (range, 50.40–51.00 Gy)	1.80 (range, 1.50–1.80)
**PET-positive LNs**	*N./a.*	*N./a.*	58.80 (range, 58.80–61.60)	2.10 (range, 1.80–2.25)
**PET-positive LR**	*N./a.*	*N./a.*	76.50 (range, 73.10–76.50)	2.25 (range, 2.00–2.25)

PB, prostate bed; LN, lymph node; LR, local recurrence; N./a., not applicable.

### Toxicity

Baseline toxicity rates are shown in **Table 3**. No significant differences were seen in the pre-RT baseline toxicity.

**Table 3:** Baseline toxicity rates of conventional salvage radiotherapy (C-SRT) and dose-escalated salvage radiotherapy (DE-SRT).

	Grade	C-SRT n = 98	DE-SRT n = 101	p
**Rectal Bleeding**	**0**	98 (100.0%)	101 (100%)	N./a.
**Proctitis**	**0**	98 (100.0%)	101 (100%)	N./a.
**Stool Incontinence**	**0**	97 (99.0%)	99 (98.0%)	1.00
	**1**	1 (1.0%)	2 (2.0%)	
**Gastrointestinal Fistula**	**0**	98 (100.0%)	101 (100%)	N./a.
**Hematuria**	**0**	98 (100.0%)	101 (100%)	N./a.
**Cystitis**	**0**	98 (100.0%)	101 (100%)	N./a.
**Urine Incontinence**	**0**	67 (68.4%)	56 (55.5%)	0.58
	**1**	26 (26.5%)	37 (36.6%)	
	**2**	5 (5.1%)	7 (6.9%)	
	**3**	0 (0.0%)	1 (1.0%)	
**Urinary Obstruction**	**0**	97 (99.0%)	101 (100.0%)	0.31
	**1**	1 (1.0%)	0 (0.0%)	
**Genitourinary Fistula**	**0**	98 (100.0%)	101 (100%)	N./a.
**Erectile Dysfunction**	**0**	28 (28.6%)	17 (16.8%)	0.11
	**1**	16 (16.3%)	15 (14.9%)	
	**2**	19 (19.4%)	17 (16.8%)	
	**3**	35 (35.7%)	52 (51.5%)	

Side effects were graded according to the Common Terminology Criteria for Adverse Events (CTCAE) version 5 (15) (N./a., not applicable).

The overall rate of early gastrointestinal toxicity ≥ grade 2 was 2.1% and 1.0% for the C-SRT and DE-SRT group, respectively. Late gastrointestinal side effects ≥ grade 2 were 1.4% and 5.3% for C-SRT and DE-SRT group. Early genitourinary toxicity ≥ grade 2 occurred in 2.1% and 3.0% of the cases for C-SRT and DE-SRT group. Late genitourinary side effects ≥ grade 2 were seen in 11.0% and 14.7% for patients with C-SRT and DE-SRT, respectively. **Table 4** shows newly occurred or worsened early (<6 months) and late (>6 months) side effects for all patients. No early gastrointestinal or genitourinary fistula was documented. One late genitourinary fistula grade 2 was reported in the DE-SRT group, whereas overall, no late gastrointestinal fistulas were seen. **Table 5** shows the newly diagnosed side effects for the subgroup of patients with C-SRT to the PB only *versus* DE-SRT of the PB with SIB. Toxicity of the remaining patients (PB+ePLNs, PB/SIB + ePLNs, PB + ePLNs/SIB, PB/SIB + ePLNs/SIB, and ePLNs/SIB) is shown in the supplementary files (see **Supplementary Table 1**).

**Table 4:** Comparison of newly diagnosed or worsened early and late toxicity rates of conventional salvage radiotherapy (C-SRT) *versus* dose-escalated salvage radiotherapy (DE-SRT) in the overall group including all patients.

	Grade	Early Toxicity Rates			Late Toxicity Rates		
		C-SRT (n = 95)	DE-SRT (n = 99)	p	C-SRT (n = 73)	DE-SRT (n = 75)	*p*
**Rectal Bleeding**	**1**	1 (1.1%)	2 (2.0%)	0.51	3 (4.1%)	6 (8.0%)	0.22
	**3**	1 (1.1%)	0 (0.0%)		0 (0.0%)	2 (2.7%)	
**Proctitis**	**1**	2 (2.1%)	2 (2.0%)	0.99	2 (2.7%)	7 (9.3%)	0.25
	**2**	1 (1.1%)	1 (1.0%)		1 (1.4%)	1 (1.3%)	
**Stool Incontinence**	**1**	0 (0.0%)	2 (2.0%)	0.16	1 (1.4%)	1 (1.3%)	0.61
	**2**	–	–		0 (0.0%)	1 (1.3%)	
**Hematuria**	**1**	0 (0.0%)	1 (1.0%)	0.33	3 (4.1%)	2 (2.7%)	0.55
	**2**	–	–		0 (0.0%)	1 (1.3%)	
**Cystitis**	**1**	3 (3.2%)	4 (4.0%)	0.74	2 (2.7%)	2 (2.7%)	0.98
**Genitourinary Fistula**	**2**	–	–	–	0 (0.0%)	1 (1.3%)	0.32
**Urine Incontinence**	**1**	17 (17.9%)	13 (13.1%)	0.62	21 (28.8%)	23 (30.7%)	0.55
	**2**	2 (2.1%)	2 (2.0%)		6 (8.2%)	6 (8.0%)	
	**3**	0 (0.0%)	1 (1.0%)		0 (0.0%)	2 (2.7%)	
**Urinary Obstruction**	**1**	1 (1.1%)	5 (5.1%)	0.11	5 (6.8%)	3 (4.0%)	0.65
	**2**	–	–		1 (1.4%)	0 (0.0%)	
	**3**	–	–		1 (1.4%)	1 (1.3%)	
**Erectile Dysfunction**	**1**	3 (3.2%)	5 (5.1%)	0.53	4 (5.5%)	3 (4.0%)	0.60
	**2**	5 (5.3%)	2 (2.0%)		5 (6.8%)	4 (5.3%)	
	**3**	12 (12.6%)	10 (10.1%)		17 (23.3%)	12 (16.0%)	

Side effects were graded according to the Common Terminology Criteria for Adverse Events (CTCAE) version 5 (15). Only patients with follow-up <6 months (n = 194) were included for analysis of early toxicity. Further, only patients with follow-up >6 months (n = 148) were included for evaluation of late toxicity.

**Table 5:** Comparison of newly diagnosed or worsened early and late toxicity rates of conventional salvage radiotherapy (C-SRT) to the prostate bed (PB) *versus* dose-escalated salvage radiotherapy (DE-SRT) to the PB and simultaneous-integrated boost (SIB) to a local recurrence.

	Grade	Early Toxicity Rates			Late Toxicity Rates		
		C-SRT PB (n = 82)	DE-SRT PB+SIB (n = 54)	p	C-SRT PB (n = 62)	DE-SRT PB+SIB (n = 40)	*p*
**Rectal Bleeding**	**1**	1 (1.2%)	1 (1.9%)	0.76	3 (4.8%)	5 (12.5%)	0.16
**Proctitis**	**1**	2 (2.4%)	1 (1.9%)	0.93	2 (3.2%)	2 (5.0%)	0.86
	**2**	1 (1.2%)	1 (1.9%)		1 (1.6%)	1 (2.5%)	
**Stool Incontinence**	**1**	0	1 (1.9%)	0.22	1 (1.6%)	0 (0.0%)	0.33
	**2**	–	–		0 (0.0%)	1 (2.5%)	
**Hematuria**	**1**	0	1 (1.9%)	0.22	3 (4.8%)	1 (2.5%)	0.39
	**2**	–	–		0 (0.0%)	1 (2.5%)	
**Cystitis**	**1**	3 (3.7%)	1 (1.9%)	0.54	1 (1.6%)	1 (2.5%)	0.75
**Urine Incontinence**	**1**	14 (17.1%)	7 (13.0%)	0.75	15 (24.2%)	13 (32.5%)	0.39
	**2**	2 (2.4%)	2 (3.7%)		6 (9.7%)	2 (5.0%)	
	**3**	–	–		0 (0.0%)	1 (2.5%)	
**Urinary Obstruction**	**1**	1 (1.2%)	2 (3.7%)	0.33	5 (8.1%)	2 (5.0%)	0.63
	**2**	–	–		1 (1.6%)	0 (0.0%)	
	**3**	–	–		1 (1.6%)	0 (0.0%)	
**Erectile Dysfunction**	**1**	3 (3.7%)	2 (3.7%)	0.90	4 (6.5%)	1 (2.5%)	0.57
	**2**	3 (3.7%)	1 (1.9%)		4 (6.5%)	1 (2.5%)	
	**3**	11 (13.4%)	6 (11.1%)		14 (22.6%)	8 (20.0%)	

Side effects were graded according to the Common Terminology Criteria for Adverse Events (CTCAE) version 5 (15). Only patients with follow-up <6 months (n=136) were included for analysis of early toxicity. Further, only patients with follow-up >6 months (n = 102) were included for evaluation of late toxicity.

### Outcome

**Figure 1:**
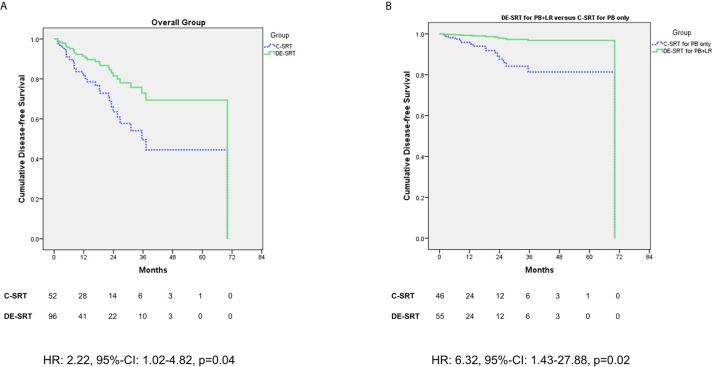
Cox regression (adjusted for the use of additive androgen deprivation therapy) of disease-free survival (DFS) for dose-escalated salvage radiotherapy (DE-SRT) *versus* conventional salvage radiotherapy (C-SRT) in the overall group **(A)** and subgroup of patients with DE-SRT for the prostate bed (PB) and local recurrence *versus* C-SRT for the PB only **(B)** (HR, hazard ratio; 95%-CI, 95%-confidence interval).

We further evaluated the outcome of patients who received DE-SRT and C-SRT. Mean DFS for C-SRT was 41.02 months (95% CI: 30.61–51.43 months) and for DE-SRT 48.12 months (41.86–54.40 months). **Figure 1** shows Cox regression of DFS of the overall group (see **Figure 1A**) and in the subgroup of DE-SRT for the elective PB and local recurrence *versus* C-SRT for PB alone (see **Figure 1B**).

**Figure 2:**
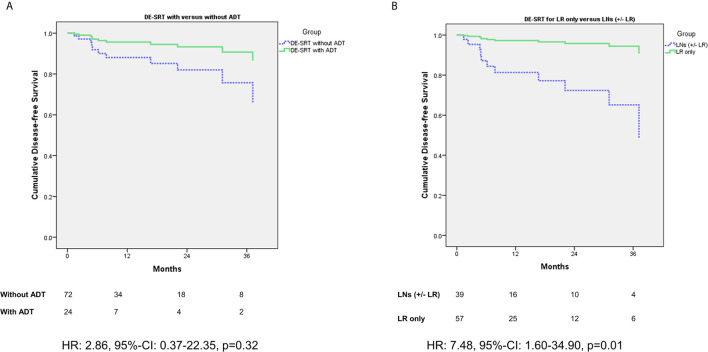
Cox regression of disease-free survival (DFS) for dose-escalated salvage radiotherapy (DE-SRT) in the subgroups of patients with/without additive androgen deprivation therapy (ADT) **(A)** and Cox regression (adjusted for use of additive androgen deprivation therapy) with respect to the PET results **(B)** (LR, local recurrence; LN, pelvic lymph node(s); HR, hazard ratio; 95%-CI, 95%-confidence interval).

**Figure 2** shows a comparison of DFS for patient with *versus* without additive ADT in the DE-SRT group (see **Figure 2A**). Further, we compared DFS of the DE-SRT group with respect to the PET results (Local recurrence only *versus* pelvic LNs and/or local recurrence, see **Figure 2B**). Moreover, we analyzed the DFS in the DE-SRT group for patients with PSA at recurrence <0.5 ng/ml *versus* ≥0.5 ng/ml. There was no significant difference (p=0.39).

We analyzed PSA response for patients who received DE-SRT and C-SRT (see **Table 6**). Overall median PSA at first follow-up was 0.07 ng/ml (range, 0.00–1.09 ng/ml) with a PSA response (≤0.2 ng/ml) of 75.0% for DE-SRT. For C-SRT the overall median PSA at first follow-up was 0.14 ng/ml (range, 0.01–51.72 ng/ml) with a PSA response of 57.5%. Overall median PSA at last follow-up was 0.07 ng/ml (range, 0.00–1.60 ng/ml),resulting in a biochemical response of 86.4% for DE-SRT. For the C-SRT group overall median PSA at last follow-up was 0.07 ng/ml (range, 0.00–1.40 ng/ml) with a PSA response of 69.6%.

**Table 6:** Outcome of dose-escalated (DE-SRT) and conventional (C-SRT) salvage radiotherapy.

	DE-SRT		C-SRT	
**PSA Response at 1. FU**				
	**Overall group**		**Overall group**	
Median PSA at 1. FU [ng/ml]	0.07 (0.00–1.09)		0.14 (0.01–51.72)	
PSA at 1. FU ≤0.2 ng/ml	75.0%		57.5%	
	**without additive ADT**	**with additive ADT**	**without additive ADT**	**with additive ADT**
Median PSA at 1. FU [ng/ml]	0.09 (0.00–1.09)	0.02 (0.00–0.96)	0.16 (0.01–51.72)	0.07 (0.05–0.07)
PSA at 1. FU ≤0.2 ng/ml	69.2%	91.3%	52.8%	100.0%
**PSA Response at last FU**				
	**Overall group**		**Overall group**	
Median PSA at last FU [ng/ml]	0.07 (0.00–1.60)		0.07 (0.00–1.40)	
PSA at last FU ≤0.2 ng/ml	86.4%		69.6%	
	**without additive ADT**	**with additive ADT**	**without additive ADT**	**with additive ADT**
Median PSA at last FU [ng/ml]	0.07 (0.00–1.60)	0.01 (0.00–0.70)	0.10 (0.00–1.40)	0.06 (0.00–0.25)
PSA at last FU ≤0.2 ng/ml	83.1%	95.7%	67.5%	83.3%

Outcome (defined by prostate-specific antigen (PSA) at first and last follow-up (FU) ≤0.2 ng/ml) of the overall group and patients with/without additive androgen deprivation therapy (ADT). Patients with admission of ADT in FU after termination of additive ADT were excluded from this endpoint.

## Discussion

The aim of this retrospective study was to compare DE-SRT and C-SRT in terms of toxicity rates. Further, we sought to report outcome data of DE-SRT. To our knowledge, this is the first study which attempted to compare DE-SRT and C-SRT. In all toxicity items (rectal bleeding, proctitis, stool incontinence, hematuria, cystitis, urine incontinence, urinary obstruction, and erectile dysfunction), no significant difference was present neither for early nor for late side effects. One late genitourinary fistula grade 2 was reported in the DE-SRT group. Overall, no gastrointestinal fistulas were seen. The outcome of DE-SRT seems good with most patients showing a PSA response at first follow-up as well as last follow-up. Patients in the overall group and in the subgroup of C-SRT to the PB *versus* DE-SRT of the PB and a local recurrence showed a significant better outcome in favor of DE-SRT.

Over the last years, the PSMA-PET has become an important diagnostic tool for patients with PC, especially in a recurrence setting. We previously reported the high clinical impact on disease staging and RT management (17). Both the impact as well as the higher diagnostic efficacy compared with other imaging techniques triggered the recommendation of PSMA-PET for patients with biochemical recurrence after prior definitive treatment in the European (18) and German (7) guidelines. With the higher sensitivity of the PSMA-PET dose escalation to specific areas became possible.

The rationale behind the dose escalation derives from the PC dose-response data. The alpha/beta ratio for PC is described to be low (19). A low alpha/beta ratio implies that the target is more resistant to low doses. Therefore, higher total doses and hypofractionated schemes for PC have been increasingly used (20, 21). In the case of SRT, the elective PB and pelvic LNs are commonly treated for microscopic disease spread with doses of 66 to 72 Gy (6, 7) and 45 to 50.4 Gy (22–24), respectively. However, keeping the low alpha/beta ratio in mind: Why should we not treat macroscopic PC in the salvage situation with the same doses as PC in the definitive situation? The European and German guideline recommend an EQD2 of 74 to approximately 80 Gy for definitive treatment of the prostate (6, 7). In our study, we used a median dose of 76.5 Gy in fractions of 2.25 Gy for a local recurrence which translates into an EQD2 (1.5 Gy) of 81.96 Gy and therefore is an appropriate dose for macroscopic PC. The guideline of the Australian and New Zealand Faculty of Radiation Oncology Genito-Urinary group (FROGG) recommends a dose escalation for local recurrence with an EQD2 of 70 to 74 Gy. Dose escalation of pelvic LNs is also recommend; however, the dose remains to be unspecified (25). For DE-SRT of LNs we used a median dose of 58.8 Gy in fractions of 2.10 Gy which translates into an EQD2(1.5Gy) of 62.16 Gy. A meta-analysis by King et al. showed that SRT doses of > 70 Gy are associated with improved relapse-free survival (26). However, most of the data originate from the pre-PSMA-PET era, and therefore, dose escalation for macroscopic tumor was barley possible.

Our data showed no increased toxicity for DE-SRT in comparison to C-SRT in the overall group as well as in the subgroup of patients with SIB to a local recurrence *versus* PB alone. Few retrospective series evaluated toxicity of PSMA-PET-based DE-SRT with a SIB to the macroscopic tumor. Schmidt-Hegemann et al. evaluated the outcome after [68Ga]PSMA-11-PET-based DE-SRT with a SIB or sequential boost with median doses of 70 Gy to the local recurrence, 60 Gy to the PB, 60.8 Gy to PET-positive LNs, and 50.4 Gy to the ePLNs (27). The authors showed acute genitourinary and gastrointestinal toxicity grade 2 in 13% and 16% of the cases, respectively. Late genitourinary and gastrointestinal toxicity grade 2 was documented in 13% and 3% (27).

Zschaeck et al. reported data of 22 patients with [68Ga]PSMA-11-PET-based DE-SRT with 66.6 Gy (1.8 Gy/fraction, EQD2(1.5Gy) = 62.79 Gy) to the PB and a SIB to local recurrences of 74 Gy (2 Gy/fraction, EQD2(1.5Gy)) = 74 Gy) to 77.7 Gy (2.1 Gy/fraction, EQD2(1.5Gy) = 79.2 Gy) (28). The ePLNs were irradiated with 54.0 Gy with a SIB of 66.0 Gy to positive LNs (28). Only 1 patient developed an acute grad 2 cystitis and diarrhea, respectively (28).

Previous series on [18F]Choline-PET-based DE-SRT showed acceptable toxicity rates as well. Wahart et al. evaluated four patients with local recurrence (29). They prescribed 62.7 Gy (1.9 Gy/fraction, EQD2(1.5Gy) = 60.91 Gy) to the PB with a SIB of 69.3 Gy (2.1 Gy/fraction, EQD2(1.5Gy) = 67.32 Gy) to the local recurrence. The authors documented no gastrointestinal toxicity ≥grade 2 and one grade 2 genitourinary toxicity (29). Fodor et al. evaluated 83 patients with LN relapse only on [11C]Choline-PET. The authors treated most of the patients with 51.8 Gy (1.85 Gy/fraction, EQD2(1.5Gy) = 49.58 Gy) to the ePLNs and a SIB with a median dose of 65.5 Gy to the LNs (30). They showed a 3-year rate of ≥ grade 2 rectal and ≥ grade 2 genitourinary toxicity of 6.6% and 26.3%, respectively (30).

The recent SAKK 09/10 evaluated the impact of dose intensified SRT for the whole PB with 64 Gy *versus* 70 Gy on toxicity and outcome. The trial showed similar acute side effects, except for a significantly greater worsening in patient-reported urinary symptoms after 70 Gy (31). However, no SIB was used in the SAKK 09/10 trial. A previous study by Cozzarini et al. evaluated the urinary toxicity for hypofractionated RT to the whole PB after RP (32). Patients with hypofractionated RT showed significantly more late urinary toxicities Grad 3/4 (18.1%) than patients with conventional fractionation (6.9%). These data predate PSMA-PET imaging and therefore a focal treatment to PET-positive areas might accomplish a survival benefit with acceptable toxicity.

PSA response and DFS showed good results for patients with PSMA-PET guided DE-SRT in our cohort of patients. This might be related to the potential of PSMA-PET localizing the site of recurrence, whereas in patients without pre-RT imaging, empiric dose planning was performed. Nevertheless, in 43.9% of the patients in the C-SRT group pre-RT PSMA-PET imaging was negative potentially including a bias. However, even with the high rate of negative PSMA-PETs in the C-SRT group the DFS is reduced which speaks in favor of dose escalation. Additionally, the patients in the DE-SRT group might benefit from a dose escalation for SRT > 70 Gy as described above and was postulated by King et al. (26).

When we stratified for additive ADT in patients with DE-SRT, patients with simultaneous hormonal deprivation showed no significant better DFS (p=0.32). However, the hazard ratio of 2.86 suggests a trend in favor of an additive ADT. This is in line with the data by Shipley et al. (33) and Carrie et al. (34) which suggest additive ADT for patients with SRT. Nevertheless, both trials did not use PSMA-PET imaging for staging before RT, but the underlying principle remains the same: ADT treats the microscopic tumor spread. However, PSMA-PET might help to identify the patients who will benefit from ADT. This should be further investigated.

When comparing sites of relapse (local recurrence only *versus* pelvic LNs and/or local recurrence), the data showed that patients with LNs exhibit a decreased DFS in comparison to patients with local relapse only. Affection of the LNs might indicate wider spread than within patients with confined disease to the PB. Such oligorecurrent patients might benefit from additional ADT (35) and therefore this topic should be further investigated.

Overall, since our data are retrospective and not powered to show superiority the results on outcome must be interpreted cautiously. The small sample size likely leads to large hazard ratios and 95% confidence intervals for the Cox regression analysis. However, the results may be understood as a hint for a better outcome for patients with PSMA-PET guided DE-SRT.

Previous studies have also shown favorable outcome for patients with PSMA-PET guided DE-SRT. Schmidt-Hegemann et al. reported that 78% of the patients reached a PSA ≤ 0.2 ng/ml after PSMA-PET guided DE-SRT (27). This is comparable to our data showing PSA response of ≤ 0.2 ng/ml of 86.4% at last follow-up. Zschaeck et al. showed a median PSA of 0.15 ng/ml at last follow-up, after a median follow-up of 29 months (28). The median PSA at last follow-up in our cohort was 0.07 ng/ml. Emmett et al. evaluated 140 patients with [68Ga]PSMA-11-PET informed SRT (36). The authors reported the outcome of patients with negative as well as positive PSMA-PET. For patients with local recurrence treatment response was 81% and for patients with LN involvement +/− local recurrence the treatment response was 38.5%. The treatment response was defined as PSA ≤ 0.1 ng/ml and a greater than 50% reduction from pre-RT PSA level. Our data confirm the reduced outcome for patients with LN involvement. Recently, Emmett et al. (37) published data of a prospective trial on [68Ga]PSMA-11-PET-based SRT in 260 patients. External beam RT as well as stereotactic body radiotherapy were allowed. Freedom from progression was defined as PSA not more than 0.2 ng/ml above the post-RT nadir. The overall 3-year freedom from progression was 64.5%, with 79% in patient with local recurrence, and 55% in patients with pelvic LNs (37). Patients with negative PSMA-PET showed the highest rates of freedom from progression with 82.5%. Recently, the EMPIRE-1 trial (38) evaluated [18F]Fluciclovine-PET for salvage RT. Patients received RT directed by conventional imaging (bone scan and CT/MRI) or by PET. The authors reported a significantly improved freedom from biochemical recurrence or persistence. Pernthaler et al. compared [18F]Fluciclovine *versus* [68Ga]PSMA-11 and showed that the overall detection rate for PC recurrence is similar with an advantage for Fluciclovine-PET in terms of local recurrence (39).

Our study has certain limitations. The median follow-up is relatively short, and a future analysis with longer follow-up is planned. Although the groups are well balanced for most factors (see **Table 1**), the retrospective cohort design of our study is a limitation. To supplement the retrospective data, only a prospective randomized controlled trial comparing patients with and without dose escalation would be helpful and therefore should be performed in the future. However, it will remain difficult to justify not performing dose-escalation in PET positive lesions. There was a significant difference in the use of PET imaging in both groups (see **Table 1**). Patients with PET are more likely to be diagnosed with the cause of PSA rise. Therefore, patients with PET are more likely to be in the DE-SRT group. There was an imbalance for coverage of the ePLNs (PB only in 86.7% in C-SRT *versus* 53.9% in DE-SRT group). However, we accounted for that by evaluating the data for the respective subgroups. In our study patients underwent PET with both [68Ga]PSMA-11 and [18F]-labeled PSMA-ligands. This might include a bias; however, this study focused on PSMA-PET-based DE-SRT and current literature indicates relative similar detection efficacy for these different PSMA-ligands (40, 41). Further, there was a significant difference concerning the admission of additive ADT in both groups (see **Table 1**). Additive ADT to SRT is based on two recent publications (33, 34). Patients in the C-SRT group received their treatment earlier than the patients in the DE-SRT group and therefore less patients with additive ADT are in C-SRT group. This might be a bias for the outcome analysis; however, we accounted for this fact by evaluating the outcome for patients with additive ADT as well as without ADT and used adjusted Cox regression analysis. Moreover, patients in the C-SRT group had a significantly shorter time from RP to RT as well as a lower PSA before SRT (see **Table 1**). To account for that, we only included patients with PSA >0.2 ng/ml at relapse for the outcome analysis.

Currently, data of a phase III trial on [68Ga]PSMA-11-PET/CT-based SRT after RP are on the way (NCT03582774). The trial compares standard SRT to PSMA-PET-based SRT. A focal dose escalation to the PSMA-positive lesions may be performed on the discretion of the treating radiation oncologist if feasible (42).

## Conclusion

PSMA-PET-based DE-SRT with SIB is feasible and showed no significantly increased toxicity rates compared with C-SRT. Further, DE-SRT showed good results in terms of PSA response and DFS. Therefore, PSMA-PET-based DE-SRT can be considered as part of the personalized cancer management of patients with PSMA-PET positive local pelvic relapse.

## Data Availability Statement

The raw data supporting the conclusions of this article will be made available by the authors, without undue reservation.

## Ethics Statement

The studies involving human participants were reviewed and approved by the institutional review board of the Technical University of Munich (TUM). Written informed consent for participation was not required for this study in accordance with the national legislation and the institutional requirements.

## Author Contributions

All authors contributed to the study conception and design. Material preparation, data collection, and analysis were performed by MV. The first draft of the manuscript was written by MV and all authors commented on previous versions of the manuscript. All authors contributed to the article and approved the submitted version.

## Conflict of Interest

ME reports an advisory role for Blue Earth Diagnostics, Point Biopharma, Telix and Janssen and patent application for rhPSMA.

The remaining authors declare that the research was conducted in the absence of any commercial or financial relationships that could be construed as a potential conflict of interest.

## Publisher’s Note

All claims expressed in this article are solely those of the authors and do not necessarily represent those of their affiliated organizations, or those of the publisher, the editors and the reviewers. Any product that may be evaluated in this article, or claim that may be made by its manufacturer, is not guaranteed or endorsed by the publisher.
