# Supplementary material for: Feasibility and Outcome of PSMA-PET-Based Dose-Escalated Salvage Radiotherapy Versus Conventional Salvage Radiotherapy for Patients With Recurrent Prostate Cancer
Source: Front Oncol. 2021 Jul 30;11:715020. doi: 10.3389/fonc.2021.715020 (PMC8362325; doi:10.3389/fonc.2021.715020)
Supplement: Supplementary file 1 [file Table_1.docx]

|  |  | **Early Toxicity Rates** | | | | | **Late Toxicity Rates** | | | | |
| --- | --- | --- | --- | --- | --- | --- | --- | --- | --- | --- | --- |
|  | **Grade** | **C-SRT**  **PB**  **+ePLNs**  **(n=13)** | **DE-SRT**  **PB/SIB**  **+ePLNs**  **(n=11)** | **DE-SRT**  **PB/SIB+**  **ePLNs/SIB**  **(n=15)** | **DE-SRT**  **PB+**  **ePLNs/SIB**  **(n=16)** | **DE-SRT**  **ePLNs/SIB**  **(n=3)** | **C-SRT**  **PB**  **+ePLNs**  **(n=11)** | **DE-SRT**  **PB/SIB**  **+ePLNs**  **(n=8)** | **DE-SRT**  **PB/SIB+**  **ePLNs/SIB**  **(n=11)** | **DE-SRT**  **PB+**  **ePLNs/SIB**  **(n=12)** | **DE-SRT**  **ePLNs/SIB**  **(n=4)** |
| **Rectal Bleeding** | **1** | 0 (0.0%) | 0 (0.0%) | 0 (0.0%) | 1 (6.3%) | 0 (0.0%) | 0 (0.0%) | 0 (0.0%) | 0 (0.0%) | 1 (8.3%) | 0 (0.0%) |
|  | **3** | 1 (7.7%) | 0 (0.0%) | 0 (0.0%) | 0 (0.0%) | 0 (0.0%) | 0 (0.0%) | 0 (0.0%) | 1 (9.1%) | 1 (8.3%) | 0 (0.0%) |
| **Proctitis** | **1** | 0 (0.0%) | 1 (9.1%) | 0 (0.0%) | 0 (0.0%) | 0 (0.0%) | 2 (3.2%) | 0 (0.0%) | 3 (27.3%) | 2 (16.7%) | 0 (0.0%) |
|  | **2** | 0 (0.0%) | 0 (0.0%) | 0 (0.0%) | 0 (0.0%) | 0 (0.0%) | 1 (1.6%) | 0 (0.0%) | 0 (0.0%) | 0 (0.0%) | 0 (0.0%) |
| **Stool Incontinence** | **1** | 0 (0.0%) | 1 (9.1%) | 0 (0.0%) | 0 (0.0%) | 0 (0.0%) | 0 (0.0%) | 0 (0.0%) | 0 (0.0%) | 1 (8.3%) | 0 (0.0%) |
| **Hematuria** | **1** | 0 (0.0%) | 0 (0.0%) | 0 (0.0%) | 0 (0.0%) | 0 (0.0%) | 0 (0.0%) | 0 (0.0%) | 1 (9.1%) | 0 (0.0%) | 0 (0.0%) |
| **Cystitis** | **1** | 0 (0.0%) | 2 (18.2%) | 1 (6.7%) | 0 (0.0%) | 0 (0.0%) | 1 (9.1%) | 0 (0.0%) | 1 (9.1%) | 0 (0.0%) | 0 (0.0%) |
| **Genitourinary Fistula** | **2** | 0 (0.0%) | 0 (0.0%) | 0 (0.0%) | 0 (0.0%) | 0 (0.0%) | 0 (0.0%) | 0 (0.0%) | 1 (9.1%) | 0 (0.0%) | 0 (0.0%) |
| **Urine Incontinence** | **1** | 3 (23.1%) | 3 (27.3%) | 0 (0.0%) | 1 (6.3%) | 2 (66.7%) | 6 (54.5%) | 1 (12.5%) | 3 (27.3%) | 4 (33.3%) | 2 (50%) |
|  | **2** | 0 (0.0%) | 0 (0.0%) | 0 (0.0%) | 0 (0.0%) | 0 (0.0%) | 0 (0.0%) | 1 (12.5%) | 2 (18.2%) | 1 (8.3%) | 0 (0.0%) |
|  | **3** | 0 (0.0%) | 0 (0.0%) | 0 (0.0%) | 0 (0.0%) | 1 (33.3%) | 0 (0.0%) | 0 (0.0%) | 0 (0.0%) | 0 (0.0%) | 1 (25.0%) |
| **Urinary Obstruction** | **1** | 0 (0.0%) | 1 (9.1%) | 1(6.7%) | 1 (6.3%) | 0 (0.0%) | 0 (0.0%) | 1 (12.5%) | 0 (0.0%) | 0 (0.0%) | 0 (0.0%) |
|  | **3** | 0 (0.0%) | 0 (0.0%) | 0 (0.0%) | 0 (0.0%) | 0 (0.0%) | 0 (0.0%) | 0 (0.0%) | 0 (0.0%) | 1 (8.3%) | 0 (0.0%) |
| **Erectile Dysfunction** | **1** | 0 (0.0%) | 1 (9.1%) | 1(6.7%) | 1 (6.3%) | 0 (0.0%) | 0 (0.0%) | 1 (12.5%) | 0 (0.0%) | 1 (8.3%) | 0 (0.0%) |
|  | **2** | 2 (15.4%) | 0 (0.0%) | 0 (0.0%) | 1 (6.3%) | 0 (0.0%) | 1 (9.1%) | 1 (12.5%) | 1 (9.1%) | 1 (8.3%) | 0 (0.0%) |
|  | **3** | 1 (7.7%) | 1 (9.1%) | 2 (13.3%) | 1 (6.3%) | 0 (0.0%) | 3 (27.3%) | 0 (0.0%) | 3 (25.0%) | 1 (8.3%) | 0 (0.0%) |

**Supplementary Table 1:** Comparison of newly diagnosed or worsened early (<6 months) and late (>6 months) toxicity rates for the subgroups: C-SRT (PB+ePLNs) and DE-SRT (PB/SIB+ePLNs, PB/SIB+ePLNs/SIB, PB+ ePLNs/SIB, and ePLNs/SIB).

Side effects were graded according to Common Terminology Criteria for Adverse Events (CTCAE) version 5 [15]. (C-SRT= conventional salvage radiotherapy, DE-SRT= dose-escalated radiotherapy, PB= prostate bed, SIB= simultaneously integrated boost, ePLNs= elective pelvic lymph nodes)
